# P-1949. The Self-Controlled Risk Interval: An Evolving Method for Vaccine Effectiveness Estimation in Complex Real-World Data

**DOI:** 10.1093/ofid/ofae631.2108

**Published:** 2025-01-29

**Authors:** Jonathan Fix, Anthony M Marchese, Hadi Beyhaghi, Matthew D Rousculp

**Affiliations:** Novavax, Inc., Gaithersburg, Maryland; Novavax, Inc., Gaithersburg, Maryland; Novavax, Inc., Gaithersburg, Maryland; Novavax, Inc., Gaithersburg, Maryland

## Abstract

**Background:**

Post-marketing assessments of vaccine effectiveness (VE) are critical to informing health policy, understanding real-world vaccine performance, and ensuring confidence in vaccination efforts, though imperfect comparator groups can limit the accuracy of VE assessments. The self-controlled risk interval (SCRI) design, a case-only analytic method historically used in safety studies, controls for time-invariant confounding using within-person comparisons and has recently been used to assess VE. This targeted review identifies and characterizes SCRI’s growing role in VE research.

**Table 1:**
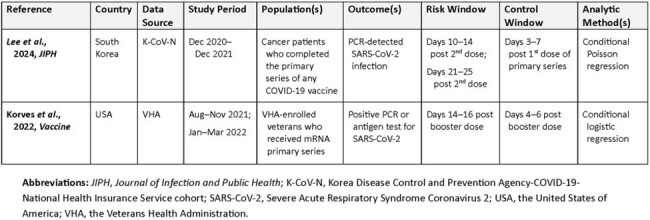
Vaccine Effectiveness Studies that Used the Self-Controlled Risk Interval Design

**Methods:**

Targeted database searches were conducted in Medline, Embase^®^, Embase Preprints, and BIOSIS Previews^®^, and the preprint servers: medRxiv, preprints with *The Lancet*, and Research Square. Key terms (and related variations) included: vaccine effectiveness, COVID-19 vaccines, and self-controlled risk interval.

**Results:**

Two VE studies that used the SCRI design were identified (Table 1). Both were retrospective database studies (one using electronic health records and the other insurance claims data), evaluated SARS-CoV-2 infection, and used similar post-vaccination control windows, but featured different post-vaccination risk windows and were conducted among notably different populations. An additional 10 identified studies included post-vaccination person-time in the comparison group but did not make within-person comparisons to address important potential confounding, acknowledging that post-vaccination benefits are not appreciated immediately. These studies varied in the study populations, outcomes assessed, comparison group composition, and analytic methods.

**Conclusion:**

Use of SCRI for VE estimation has been limited; however, the increasing inclusion of post-vaccination control arm person-time in other COVID-19 VE studies merits the broadening of its application. The results of this review suggest that, in the context of COVID-19, SCRI VE evaluation is a promising tool where there are no suitable comparison groups, increasingly complex individual backgrounds (e.g., infection history, number and timing of doses, or heterologous vaccination), or insufficient information for confounding control.

**Disclosures:**

Jonathan Fix, PhD, Novavax, Inc.: employee|Novavax, Inc.: Stocks/Bonds (Public Company) Anthony M. Marchese, PhD, Novavax Inc: Employee|Novavax Inc: Stocks/Bonds (Public Company) Hadi Beyhaghi, MD, PhD, Novavax, Inc.: employee|Novavax, Inc.: Stocks/Bonds (Public Company) Matthew D. Rousculp, PhD, Novavax, Inc.: employee|Novavax, Inc.: Stocks/Bonds (Public Company)

